# Electroconvulsive therapy combined with lithium developed reversible pure anomic aphasia: a case report

**DOI:** 10.1186/s12888-022-04323-1

**Published:** 2022-10-27

**Authors:** Qian Yang, Xiaofei Cheng, Zhewei Su, Linyuan Sun, Mingli Li

**Affiliations:** 1 Mental Health Center, West China Hospital, Sichuan University, No.28 Dianxin South Road, Chengdu, 610041 China; 2 Sichuan Clinical Medical Research Center for Mental Disorders, Chengdu, China

**Keywords:** Electroconvulsive therapy, Lithium, Pure anomic aphasia, Bipolar disorder, Case report

## Abstract

**Background:**

Electroconvulsive therapy (ECT) combined with mood stabilizers is an effective method of treatment for manic episodes; however, there are controversial views on its side effects.

**Case presentation:**

A 53-year-old man was diagnosed with bipolar disorder during a manic episode, and had previous conditions such as hypertension, and diabetes. He developed reversible delirium and anomic aphasia during combined treatment with lithium and ECT (Li-ECT). No other neurological symptoms or signs happened during the one-month follow-up period.

**Conclusions:**

The anomic aphasia appeared after ECT was reversible. Li-ECT should be administered with caution to middle- and older-aged patients with comorbidities, and serum Li levels should be closely monitored during the treatment period.

## Background

Bipolar disorder (BD) is a serious illness characterised by intermittent mania/hypomania and depression. Lithium (Li) is a mood stabilizer widely used in its treatment. In some cases, electroconvulsive therapy (ECT) is recommended as a highly and rapidly effective treatment for the management of serious acute manic episodes [1]. Although some studies have reported that Li and ECT (Li-ECT) combined treatment is safe and more effective than either Li or ECT alone for both depression [2] and mania [3], opposing views have implied that Li-ECT may increase the risk of cognitive impairment and delirium [4, 5]. Here, we report a case of BD where the patient developed delirium and reversible pure anomic aphasia after Li-ECT treatment.

## Case presentation

A 53-year-old man had been diagnosed with major depressive disorder 13 years previously and had been treated with venlafaxine 150 mg/day for 3 years, during which time his mood was stable. Five years prior to admission, he had been diagnosed with BD, as he experienced a manic episode with irritability, agitation, inappropriate elation, markedly increased energy and activity levels, poor judgment, and grandiose delusions. He recovered through treatment with olanzapine 20 mg/d and Li 500 mg/d combined with five sessions of ECT without significant side effects, except for reversible short-term memory loss. During the 5 years, he was hospitalised twice, once for an episode of depression and once for an episode of mania. On admission, he manifested excitement, loquaciousness, irritability, increased activity levels, decreased need for sleep, increased spending, and abusive behaviour; he had attacked family members and had experienced grandiose delusions for 1 week. The Young Mania Rating Scale score was 45.

He had hypertension (grade 2) for 7 years, and his blood pressure, which ranged from 110–140/60–90 mmHg, was controlled using amlodipine besylate 5 mg/d. He had also been diagnosed with diabetes at 4 years prior to admission, and metformin 1500 mg/d was used to control his blood glucose levels. He had abused alcohol for 20 years but had stopped drinking for the last 13 years. Both his father and younger brother had major depressive disorder.

The vital-sign (heart rate, respiratory function, blood pressure, and neurological assessment) as well as chest and head computed tomography (CT) examinations revealed no obvious abnormalities. Other blood-test results, including of thyroid function, electrolytes, cortisol, and liver and kidney function were normal, except for creatine kinase (429 IU/L; reference range, 19–226 IU/ L).

He was treated with Li 250 mg twice a day, and intramuscular injections of haloperidol 10 mg twice a day for 3 days, but still experienced extreme aggression with impulsive speech and violent behaviour, including damaging the nurses’ station. Li-ECT was then started as treatment. ECT with bilateral temporal electrode placement was administered every day for the first 3 days, followed by every-other-day administration, for a total of nine sessions over 3 weeks. ECT was performed using the Thymatron System IV (America Somatics LLC, Lake Bluff, IL, USA). All nine ECT sessions were conducted under anaesthesia (penehyclidine hydrochloride 0.4 mg, propofol 80 mg, succinylcholine 40–50 mg). Stimulation parameters were adjusted as follows: 900 mA current, 6.3–7.6 s stimulus duration, the stimulus dose was set from 40 to 65% (40% for the first two treatment sessions, 45%, 50%, 55%, 60% for the four middle sessions, and 65% for the final three sessions). Motor seizure durations ranged from 17 to 104 s during the course of the ECT (33 s, 50 s and 36 s for the final three sessions, respectively). Seizure adequacy was defined as follows: seizure lasting for more than 20 s, inhibition index being greater than 80%, energy index being greater than 8000UV, along with slight spasticity and rigidity of limbs and mouth occlusion.

After the first three ECT sessions, we increased the Li dosage to 500 mg, twice a day, because poor symptom control and extreme aggression persisted.

**Fig. 1:**
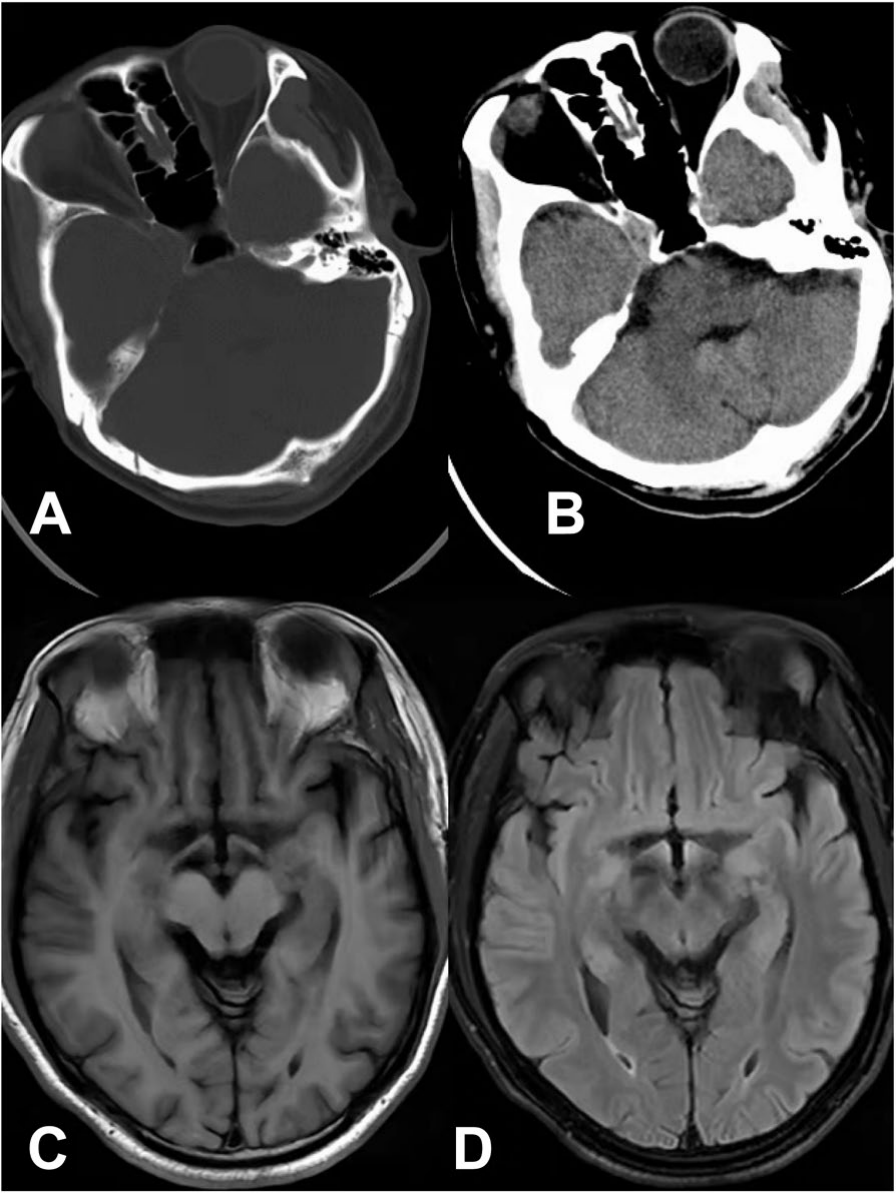
**A** and **B** show the head computed tomography scan of the patient; **C** and **D** show the head magnetic resonance imaging scan of the patient

After the ninth ECT session, the patient developed delirium with disorientation of time and location, short-term memory decline, dull and slow expressions, easy distraction, sleepiness, auditory and visual hallucinations. Confusion Assessment Method [6] was used to confirm delirium diagnosis, and the score was 29. His blood Li concentration was 1.21 mmol/L. ECT was discontinued, and the Li dosage was decreased to 500 mg/d. During ECT, his blood pressure fluctuated between 102–155/67–108 mmHg. Five days later, his consciousness was recovered, however he developed a new symptom, anomic aphasia (word selection failure). His spontaneous speech was fluent, but his naming was notably impaired. For example, he knew that a doctor was involved in medical treatment but could not say the word ‘doctor’; he knew that the doctor's job card was ‘something that can identify what type of work the person is doing’ but could not say the words ‘job card’; he knew that the pen was used to write but could not say the word ‘pen’. Despite his word-finding difficulty, his understanding of written and spoken language remained unaltered, and he could write and read accurately. Anomic aphasia was confirmed by the Chinese Rehabilitation Research Center Standard Aphasia Examination [7]. However, no other neuropsychological assessment was used to evaluate the patient's additional cognitive functions. Brain CT revealed no bleeding or ischaemia (Fig. 1 A and B). The patient also underwent head magnetic resonance imaging (MRI) 3 days after the appearance of anomic aphasia, and no abnormalities were identified (Fig. 1 C and D). Within a week, his anomic aphasia disappeared and his mood was stable. The patient was subsequently prescribed Li 750 mg/d and quetiapine 200 mg/d. On his first review visit one-month later, his mind was clear, his orientation was intact, and he was able to name objects correctly. He reported that his mood had been stable. The score of Young Mania Rating Scale was 5, the score of Hamilton Depression Rating Scale -17 was 6, the score of Positive and Negative Syndrome Scale was 40.

## Discussion and conclusions

Here, we report a rare case of a patient diagnosed with BD who was treated with Li-ECT and developed delirium followed by reversible anomic aphasia.

The patient had no fever or movement disorders, and head CT/MRI showed no obvious abnormalities. Stroke, head trauma, head infection, and other organic brain diseases were excluded. We presume that the acute brain dysfunction associated with delirium may have been caused by the Li-ECT treatment. There are controversial views regarding the combined use of Li and ECT. Some studies have proposed that Li-ECT is effective and safe [2, 3, 8]. Conversely, other studies have suggested that the coadministration of Li and ECT should be implemented with caution considering its potential side effects such as delirium, cognitive impairment [4, 9], prolonged or tardive seizures, and serotonin syndrome [10], usually associated with high serum Li concentrations [11, 12]. The incidence of delirium associated with Li-ECT treatment has been reported to be 5–10% [8, 13]. In this case, although the patient received a relatively low dosage of Li, 1000 mg/d, during ECT, his serum Li concentration was 1.21 mmol/L, and delirium developed. This indicates that, in clinical settings, the serum Li concentration should be closely monitored to prevent side effects during the course of Li-ECT treatment for BD. In addition, the Li dosage should be appropriately reduced during ECT [14], and it is preferable to maintain serum Li levels at lower values than the therapeutic range.

The patient had received five sessions of ECT combined with olanzapine 20 mg/d and Li 500 mg/d 5 years previously, but without obvious side effects. However, delirium and pure anomic aphasia occurred during the most recent Li-ECT treatment. The possible reasons are the following: first, the patient had previous hypertension for 7 years and diabetes for 4 years, which are high-risk factors for a vulnerability of brain microvessels, which might lead to greater brain-function fragility compared to healthy people. Indeed, patients with previous organic brain disorders were reported to be more likely to develop delirium during the course of Li-ECT [15]. Second, the patient received ECT every other day, which is a relatively high-frequency treatment. A previous study indicated that higher ECT frequency was related to confusional states [16]. Third, the intensity of ECT was slightly higher in the last four sessions, reaching 65%, and the seizure duration lasted 33-50 s. A higher stimulus dose was found to be associated with increased cognitive side effects [17]. Forth, ECT does not seem to develop cognitive impairment in depressed elderly patients that are physically healthy [18], but those with comorbidities, such as dementia, cerebrovascular or Parkinson's disease, were more prone to cognitive side effects after ECT [19–21]. In this regard, it has been suggested that for middle-aged and older adult patients with comorbidities, the intensity and frequency of ECT should be personalised and closely monitored.

After recovery of consciousness, the patient showed symptoms of anomic aphasia. Anomic aphasia often results from lesions of the posterior left middle temporal gyrus or temporo-occipital junction [22], such as infarction, haemorrhage, and tumours [22, 23]. However, the neuroimaging examination of the patient did not reveal significant abnormalities. We speculated that the aphasia might be related to the ECT. The electrodes were placed bilaterally, and the ECT intensity was relatively high. This might have caused spasm of small blood vessels, resulting in inadequate blood perfusion and transient brain dysfunction [24–26]. Another possible mechanism of aphasia after ECT was suggested to be a variation of Todd´s postictal paralysis or a prolonged, focal seizure that was not registered via EEG electrodes routinely used in ECT [24, 27, 28]. While, it should be noted that previous cases reported transient aphasia occurred after Li toxicity, and disappeared after rehydration and Li withdrawal [29, 30]. The blood Li concentration of our patient was 1.21 mmol/L. Although it was not a toxic concentration, the role of Li in the development of anomic aphasia in the case cannot be ruled out.

In our patient, ECT was discontinued after the appearance of delirium. The anomic aphasia arose after ECT and was reversible in our patient who had no notable brain lesions. Reversible anomic aphasia could not be a contraindication of ECT, when the potential benefit from subsequent ECT treatments outweighs the risk of developing it. However, Li-ECT should be administered with caution to middle-aged and older adult patients with comorbidities in order to prevent this side effect. Moreover, the serum Li concentration should be closely monitored during Li-ECT treatment.

## Data Availability

The raw data is available from the corresponding author on reasonable request.

## Abbreviations

CT: Computed tomography
Li: Lithium
ECT: Electroconvulsive therapy
Li-ECT: Lithium combined with electroconvulsive therapy
MRI: Magnetic resonance imaging
